# Comprehensive RNA sequencing and co-expression network analysis to complete the biosynthetic pathway of coumestrol, a phytoestrogen

**DOI:** 10.1038/s41598-018-38219-6

**Published:** 2019-02-13

**Authors:** Jungmin Ha, Young-Gyu Kang, Taeyoung Lee, Myoyeon Kim, Min Young Yoon, Eunsoo Lee, Xuefei Yang, Donghyun Kim, Yong-Jin Kim, Tae Ryong Lee, Moon Young Kim, Suk-Ha Lee

**Affiliations:** 1 Department of Plant Science and Research Institute of Agriculture and Life Sciences, Seoul National University, Seoul, Republic of Korea; 2 Plant Genomics and Breeding Institute, Seoul National University, Seoul, Republic of Korea; 3 Basic Research & Innovation Division, R&D Center, AmorePacific Corporation, Yongin-si, Gyeonggi-do, Republic of Korea

## Abstract

Coumestrol (CMS), a coumestan isoflavone, plays key roles in nodulation through communication with rhizobia, and has been used as phytoestrogens for hormone replacement therapy in humans. Because CMS content is controlled by multiple genetic factors, the genetic basis of CMS biosynthesis has remained unclear. We identified soybean genotypes with consistently high (Daewonkong) or low (SS0903-2B-21-1-2) CMS content over 2 years. We performed RNA sequencing of leaf samples from both genotypes at developmental stage R7, when CMS levels are highest. Within the phenylpropanoid biosynthetic pathway, 41 genes were tightly connected in a functional co-expression gene network; seven of these genes were differentially expressed between two genotypes. We identified 14 candidate genes involved in CMS biosynthesis. Among them, seven were annotated as encoding oxidoreductases that may catalyze the transfer of electrons from daidzein, a precursor of CMS. Two of the other genes, annotated as encoding a MYB domain protein and a MLP–like protein, may increase CMS accumulation in response to stress conditions. Our results will help to complete our understanding of the CMS biosynthetic pathway, and should facilitate development of soybean cultivars with high CMS content that could be used to promote the fitness of plants and human beings.

## Introduction

Plants synthesize secondary metabolites to promote their own survival, and some of these compounds have antioxidant or antibiotic effects^1,2^. Several lines of evidence suggest that plant secondary metabolites, especially isoflavones, can improve the fitness of both humans and plants. Accordingly, a great deal of research has been conducted on isoflavones. Legume species, including the economically important crop plant soybean, are rich in isoflavones with estrogenic and antioxidant functions^3,4^. Within plants themselves, isoflavones play crucial roles in nodulation and nitrogen fixation^5^ and defense against environmental stresses^6^. In the context of human health, isoflavones, as phytoestrogens, can decrease the risk of menopausal symptoms, breast cancer, osteoporosis, dementia, and cardiovascular disease^7–13^.

Declining estrogen levels in postmenopausal women are associated with a variety of cutaneous changes, including dryness, wrinkling, poor healing, and hot flashes, many of which can be improved by estrogen supplementation^14^. However, the estrogens used in hormone replacement therapy can promote the initiation and progression of breast cancer^15–17^. The effects of estrogen are mediated by two estrogen receptors (ERs), ERα and ERβ, which are distributed differently in each tissue^18,19^. ERα mediates the breast cancer–promoting effects of estrogens, whereas ERβ inhibits breast cancer cell proliferation and tumor formation^20^. Therefore, it has been proposed that dietary or synthetic ERβ-selective estrogens would lack the breast cancer–promoting properties of the estrogens used in hormone replacement regimens^20^.

Coumestrol (CMS), a coumestan isoflavone, is the most abundant polyphenol in soybean leaves^21^ and functions as a phytoestrogen that is structurally and functionally similar to 17β-estradiol, an estrogen steroid hormone^22–25^. ER-binding assays revealed that CMS has a 15-fold higher binding affinity for ERβ than for ERα^26^. Intake of CMS is associated with reduced risk of breast cancer^27^; in addition, CMS prevents skin photoaging by suppressing FMS-like tyrosine kinase 3, which causes collagen degradation and skin wrinkling^28^. CMS can decrease melanin synthesis, which darkens the skin, as well as alleviate symptoms caused by excessive melanin synthesis, such as melisma, solar lentigo, dark spots, and freckles^29^. In light of these health benefits, CMS has been suggested as a promising dietary supplement that could prevent disease and improve the health of postmenopausal women.

CMS is a soybean phytoalexin that is present in soybean leaves and roots, rather than seeds, and CMS content varies depending on environmental conditions and growth stage^21,30,31^. CMS accumulates to high levels after drought stress in root, doubling the extent of mycorrhizal colonization^32,33^. Because other isoflavones, such as daidzein and formononetin, are involved in signaling in rhizosphere plant-microbe interactions, CMS has been implicated in drought tolerance in legumes, an effect mediated through communication with mycorrhiza^33–35^.

CMS is derived from the soybean isoflavone daidzein, via dihydrodaidzein and 2’-hydroxydaidzein, through two biosynthetic pathways that remain incompletely understood^36,37^. Because many environmental factors affect the biosynthesis and accumulation of isoflavones, and epistatic interactions among multiple QTLs with small individual effects are responsible for a large proportion of the variation, it has been very challenging to elucidate the genetics governing isoflavone biosynthesis^38–41^. Due to the important implications of soybean isoflavones and phytoalexins for plant defense and human health, it would be valuable to identify the enzymes responsible for CMS biosynthesis from its precursor, daidzein. Knowledge of these enzymes would facilitate successful manipulation of CMS levels *in planta*.

In this study, we measured the CMS contents of 31 soybean genotypes, and selected those with consistently high or low CMS contents for gene expression profiling. To shed light on the genetics of CMS biosynthesis, comprehensive RNA sequencing (RNA-seq) was conducted on the leaf tissues at growth stage R7, when CMS levels are highest^42^. Based on the differentially expressed genes (DEGs) between high-CMS and low-CMS genotypes, as well as their functional co-expression network, we identified candidate genes involved in biosynthesis of CMS from daidzein. Our results provide a set of target genes for manipulations aimed at increasing CMS levels in soybean cultivars, with the goal of improving the welfare of menopausal women from clinical and cosmetic perspectives.

## Results

### CMS contents in soybean genotypes

**Figure 1:**
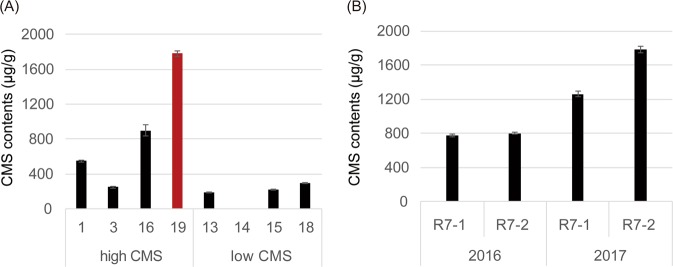
Measurements of CMS content. (**A**) CMS contents in eight selected soybean genotypes with three replications. Nineteen (Daewonkong, red bar) and fourteen (SS0903-2B-21-1-2, blue bar) exhibited the highest and the lowest CMS contents in 2017, respectively. Error bars indicate standard deviation. (**B**) CMS contents in Daewonkong. The contents were measured at two different time points in each of the subsequent years (2016 and 2017), for all three replications. The CMS contents of Daewonkong leaf samples at growth stage R7 were measured on October 6 (R7-1, 2016) and 13 (R7-2, 2016) in 2016 and September 26 (R7-1, 2017) and October 10 (R7-2, 2017) in 2017. Error bars indicate standard deviation.

We measured CMS contents in leaf samples from 31 soybean genotypes collected at growth stage R7 in 2016; three replicates were performed for each genotype (Supplementary Fig. 1). CMS content varied from 0 to 1,650.55 μg/g. Among the 31 cultivars tested, four with high CMS content, Chamame (1,650.55 μg/g), Geomjeongsaeol (1,209.70 μg/g), SG-257 (1,173.11 μg/g), and Daewonkong (802.08 μg/g), and four with low CMS content, SS0903-2B-21-1-2 (30.32 μg/g), Haepum (39.79 μg/g), SS0905-2B-179-1-1 (48.98 μg/g), and Sinhwa (142.90 μg/g), were selected for repeated cultivation and testing. CMS contents were measured in 2017, again in three replicates, to verify the results from 2016 (Fig. 1A). Daewonkong and SS0903-2B-21-1-2 had the highest (1781.79 μg/g) and the lowest (3.81 μg/g) CMS contents, respectively, in 2017. These two genotypes with consistently high and low CMS contents for two years were further investigated by RNA-seq to reveal the genetics underlying CMS biosynthesis.

### RNA-seq and DEG profiling

**Figure 2:**
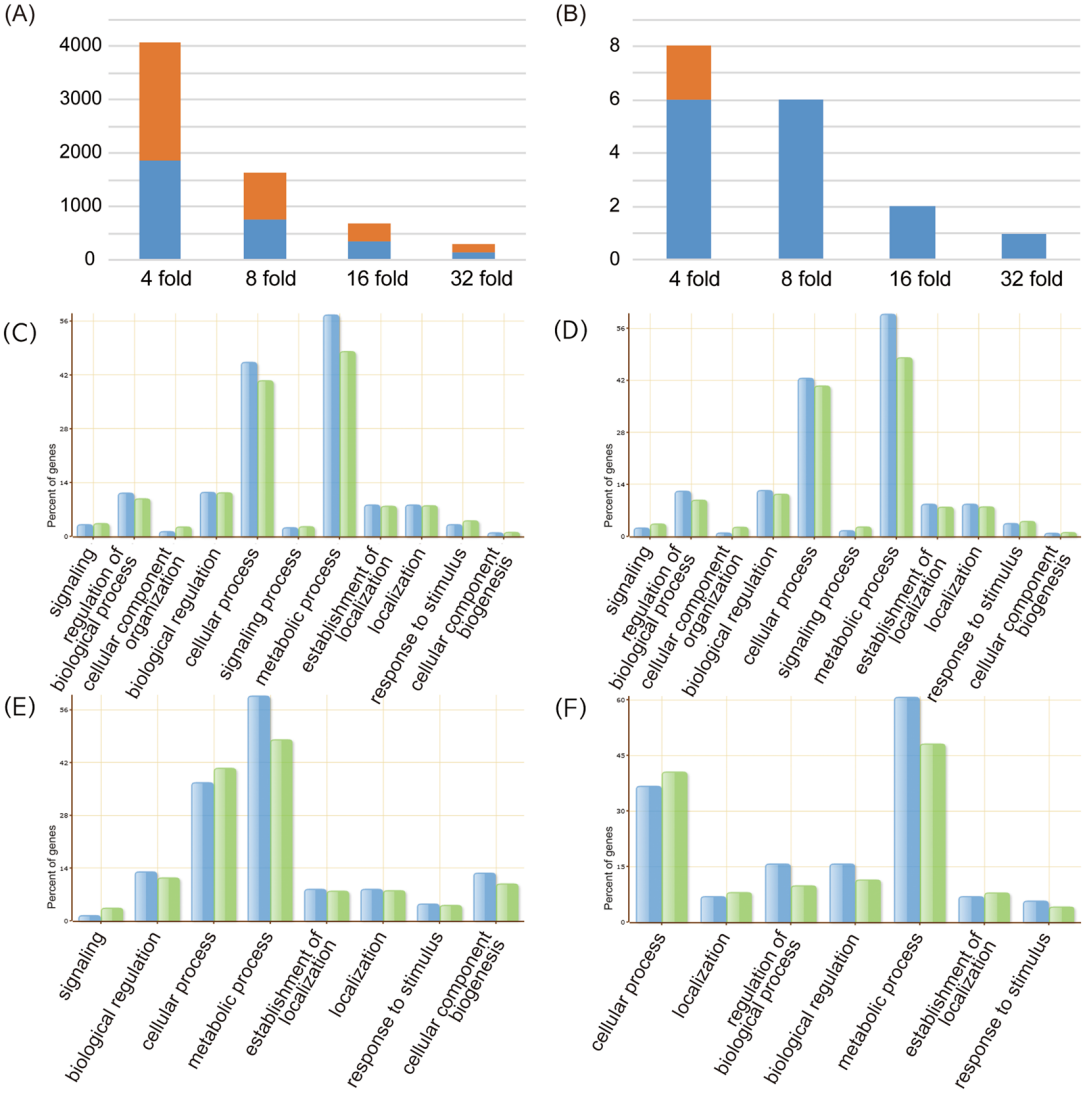
Number of DEGs and GO enrichment analysis. (**A**) The numbers of DEGs detected between Daewonkong and SS903-B2-21-1-2. The DEGs up-regulated in Daewonkong and SS0903-B2-21-1-2 are indicated in blue and orange, respectively. Each column indicates the numbers of DEGs with 4-, 8-, 16-, and 32-fold changes, respectively, between Daewonkong and SS0903-B2-21-1-2. (**B**) The number of DEGs in the network of phenylpropanoid biosynthesis. GO enrichment of (**C**) 4-, (**D**) 8-, (**E**) 12-, and (**F**) 32-fold DEGs between Daewonkong and SS0903-B2-21-1-2. Green bar indicates background reference, and blue bar indicates the query of the DEGs.

To identify the differences in gene expression involved in phenylpropanoid biosynthesis between Daewonkong and SS0903-2B-21-1-2, we extracted total RNA from leaf tissues at developmental stage R7. A total of 237 and 245 million reads, encompassing 24 and 25 Gb, respectively, were generated per genotype and about 70% and 75% of total reads were properly mapped to the *G. max* reference genome sequence (www.phytozome.net/soybean)^43^ (Supplementary Table 1). Comparison of expression levels between Daewonkong and SS0903-2B-21-1-2 revealed 4,046 DEGs with FC value of at least 4 (1,629 DEGs with FC 8, 684 DEGs with FC 16 and 283 DEGs with FC 32) and, overall about 50% of DEGs were up-regulated in Daewonkong (45.7–52.6%) (Fig. 2A). The DEGs were annotated against the AgriGO genome locus background, and metabolic process was the most enriched GO term in all four DEG sets (Fig. 2C–F). KEGG pathway analysis revealed that ~40% and ~30% of DEGs were assigned to metabolic pathways and biosynthesis of secondary metabolites, respectively (Supplementary Fig. 2). These results indicate that the secondary metabolic pathways are differentially regulated between the two genotypes.

### The expression of genes in the phenylpropanoid biosynthesis pathway

**Figure 3:**
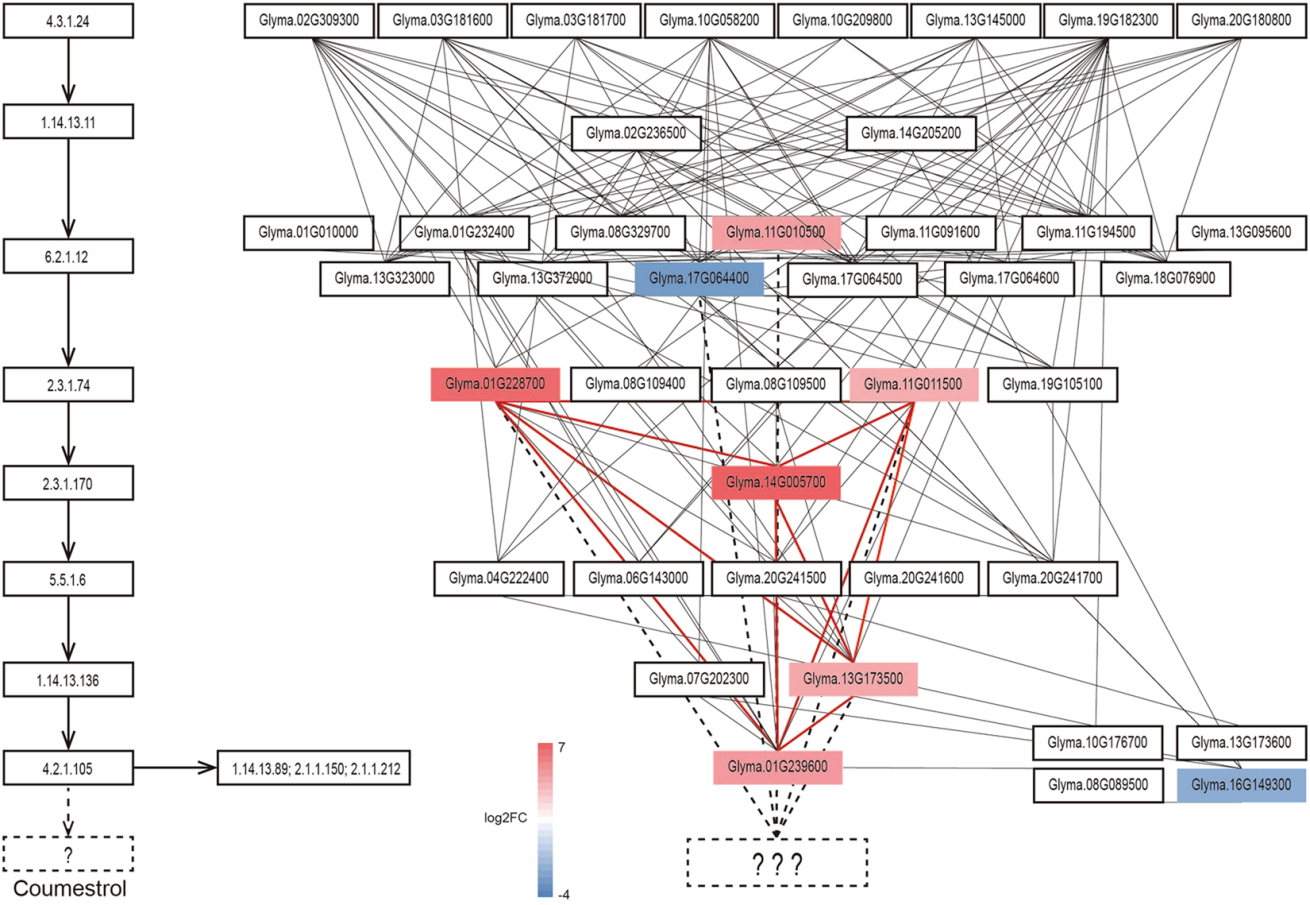
Soybean homologs in the network of the CMS biosynthesis pathway. EC numbers of key enzymes involved in biosynthetic pathways from phenylalanine to CMS are indicated in the boxes on the left. Dotted lines and rectangles indicate unknown pathways and enzymes. Homologous genes corresponding to each EC number are located on the right in the same row. DEGs up- and down-regulated in Daewonkong are indicated in red and blue, respectively. Connections in the co-expression network connecting the DEGs are highlighted by red lines. The color scale on the bottom indicates log_2_FC value.

To characterize differences in the expressions of genes involved in phenylpropanoid biosynthesis, we searched KEGG (http://www.genome.jp/kegg/) for soybean homologs of key enzymes in this pathway. In the biosynthetic pathway of phenylpropanoid, starting from phenylalanine to daidzein and its byproducts (Supplementary Fig. 3), we identified 72 candidate soybean homologs. Among them, 41 were connected with each other in a functional co-expression network in the SoyNet database (http://www.inetbio.org/soynet/) (Fig. 3)^44^. Eight of the genes in this network were differentially expressed between Daewonkong and SS0903-2B-21-1-2, and thus represent the DEGs most likely to affect the content of CMS, an end product of isoflavone biosynthesis (Supplementary Table 3). Two of these genes, Glyma.16G149300 (LOC100811727) and Glyma.17G064400 (LOC100779668), were down-regulated in Daewonkong (log_2_FC −2.7 and −3.1), whereas the other six, Glyma.01G228700 (chalcone synthase, CHS7), Glyma.01G239600 (2-hydroxyisoflavanone dehydratase, HIDH), Glyma.11G010500 (4-coumarate:CoA ligase, 4CL13), Glyma.11G011500 (CHS8), Glyma.13G173500 (isoflavone synthase, IFS2), and Glyma.14G005700 (chalcone reductase, CHR14), were up-regulated (up to log_2_FC 6.5). Five of the up-regulated genes were tightly connected with each other in the co-expression network of the phenylpropanoid biosynthesis pathway (Fig. 3, Supplementary Fig. 4). Among the eight DEGs, seven in the upstream pathway of CMS biosynthesis (i.e., all except Glyma.16G149300) were used to predict candidate genes for the unknown pathway (Fig. 3).

### Identification of candidate genes for CMS biosynthesis

To identify candidate genes for biosynthesis of CMS from daidzein, we applied three prediction approaches (Supplementary Table 4). First, using the 41 genes in the network as guide genes (Fig. 3), we searched the whole soybean gene network of 40,812 genes for genes closely connected to the guide genes (“guide prediction”). Second, the genes closely connected to the seven DEGs involved in phenylpropanoid biosynthesis were identified using the same network. Third, candidate genes were predicted in the context of subnetworks consisting of central hubs and their neighbors, and connections between the hub genes and the seven DEGs were identified (“hub prediction”) (Supplementary Table 4)^44^. We then listed the top 20 genes predicted from each approach, along with their GO terms from three different databases. The three methods identified 3, 11, and 9 genes as DEGs, all of which were up-regulated in Daewonkong (Supplementary Fig. 5). Overall, 14 genes were predicted from the three prediction approaches, of which seven were identified by two or more approaches (Supplementary Table 5). These 14 candidate genes may play a key role in determining CMS contents in soybean.

### Validation of gene expression level by qRT-PCR

**Figure 4:**
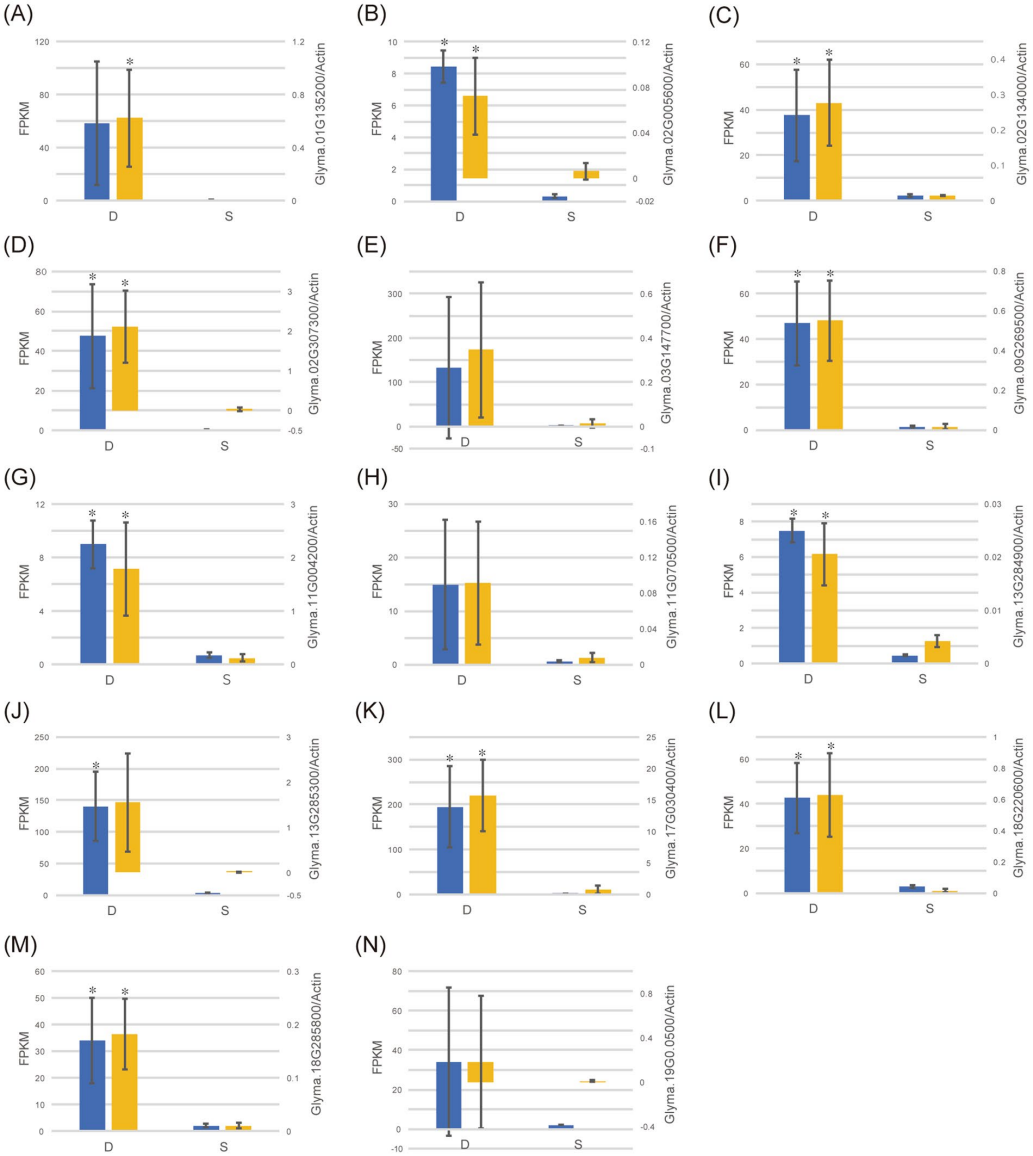
Validation of RNA-seq results by qRT-PCR. Left and right y-axes indicate FPKM values from RNA-seq (blue bar) and relative transcript abundance from qRT-PCR (orange bar). D and S on the x-axis represent Daewonkong and SS0903-2B-21-1-2, respectively. Bars indicate means and standard deviation of three biological replicates. Asterisk above each bar indicates statistical difference between genotypes, as determined by Student’s t-test (*p* < 0.05). (**A**) Glyma.01G135200, cytochrome P450, family 82, subfamily C, polypeptide 4. (**B**) Glyma.02G005600, myb domain protein 15. (**C**) Glyma.02G134000, carboxyesterase 13. (**D**) Glyma.02G307300, NAD(P)-linked oxidoreductase superfamily protein. (**E**) Glyma.03G147700, disease resistance–responsive (dirigent-like protein) family protein. (**F**) Glyma.09G269500, NAD(P)-binding Rossmann-fold superfamily protein. (**G**) Glyma.11G004200, alpha/beta-Hydrolases superfamily protein. (**H**) Glyma.11G070500, NmrA-like negative transcriptional regulator family protein. (**I**) Glyma.13G284900, organic cation/carnitine transporter4. (**J**) Glyma.13G285300, cytochrome P450, family 82, subfamily C, polypeptide 4. (**K**) Glyma.17G030400, MLP-like protein 423. (**L**) Glyma.18G220600, NAD(P)-binding Rossmann-fold superfamily protein. (**M**) Glyma.18G285800, NAD(P)-linked oxidoreductase superfamily protein. (**N**) Glyma.19G030500, HXXXD-type acyl-transferase family protein.

We validated the expression levels of the 14 DEGs by quantitative reverse-transcription (qRT) PCR (Fig. 4). The qRT-PCR results were consistent with the RNA-seq data: all 14 DEGs up-regulated in Daewonkong in the RNA-seq data were also up-regulated in the qRT-PCR results (Supplementary Fig. 5).

### Proposed model for the biosynthetic pathway from Daidzein to CMS

**Figure 5:**
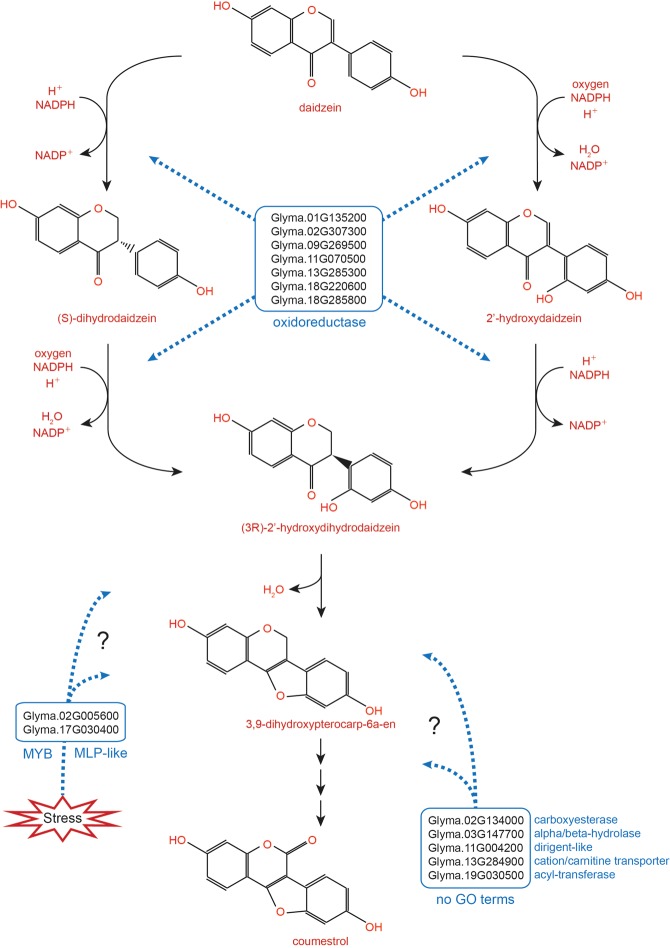
Candidate genes involved in the biosynthetic pathway from daidzein to CMS. The lists of genes in the blue boxes indicate the candidate genes involved in the pathways of CMS biosynthesis. The blue box at the top includes Glyma.01G135200, AT4G31940.1, cytochrome P450, family 82, subfamily C, polypeptide 4; Glyma.02G307300, AT1G59960.1, NAD(P)-linked oxidoreductase superfamily protein; Glyma.09G269500, AT2G45400.1, NAD(P)-binding Rossmann-fold superfamily protein; Glyma.11G070500, AT4G39230.1, NmrA-like negative transcriptional regulator family protein; Glyma.13G285300, AT4G31940.1, cytochrome P450, family 82, subfamily C, polypeptide 4; Glyma.18G220600, AT2G45400.1, NAD(P)-binding Rossmann-fold superfamily protein; and Glyma.18G285800, AT1G59960.1, NAD(P)-linked oxidoreductase superfamily protein. The blue box on the bottom left includes Glyma.02G005600, AT3G23250.1, myb domain protein 15; and Glyma.17G030400, AT1G24020.1, MLP-like protein 423. The blue box on the bottom right includes Glyma.02G134000, AT3G48700.1, carboxyesterase 13; Glyma.03G147700, AT5G42500.1, disease resistance–responsive (dirigent-like protein) family protein; Glyma.11G004200, AT1G47480.1, alpha/beta-Hydrolases superfamily protein; Glyma.13G284900, AT3G20660.1, organic cation/carnitine transporter4; and Glyma.19G030500, AT5G39090.1, HXXXD-type acyl-transferase family protein.

Based on guide prediction and hub prediction approaches, 14 DEGs were predicted to be involved in CMS biosynthesis (Supplementary Table 5). All 14 were highly up-regulated in Daewonkong, and qRT-PCR results were in close agreement with the RNA-seq data. These genes were mapped against three GO databases: agriGO, AtgO, and Uniprot-GO. Of the 14, four (Glyma.02G307300, Glyma.09G269500, Glyma.11G070500, and Glyma.18G220600) were mapped to steroid and flavonoid biosynthetic processes; two (Glyma.02G005600 and Glyma.17G030400) were related to stress responses; and eight genes were not previously mapped in any GO database. The biosynthetic reactions from daidzein to (3 R)-2′-hydroxydihydrodaidzein comprise a series of oxidations (NADPH → NADP^+^) and hydrolysis (Fig. 5)^36,37^. All four DEGs involved in steroid and flavonoid biosynthetic processes (Glyma.02G307300, Glyma.09G269500, Glyma.11G070500, and Glyma.18G220600), as well as Glyma.18G285800 (GO-unmapped), encode proteins that catalyze NAD(P) oxidation/reduction reactions. NAD(P)-linked oxidoreductases (Glyma.02G307300 and Glyma.18G285800) catalyze the transfer of electrons from one molecule to another using NADPH or NADP^+^ as a cofactor, and the NAD(P)-binding Rossmann fold (Glyma.09G269500, Glyma.11G070500, and Glyma.18G220600) is involved in catalysis of NAD(P)-dependent oxidation^45,46^. The NmrA-like protein encoded by Glyma.11G070500 contains two domains, including a Rossmann fold^47^. Therefore, we speculate that the proteins encoded by these seven genes catalyze the NADPH oxidation reactions starting from daidzein (Fig. 5).

## Discussion

Soybean is one of the most important crops in the world due to its high production of protein and oil. In addition, it is a valuable nutraceutical ingredient because it contains several phytochemicals, including isoflavones, saponins, phenolic acids, and linoleic acids. In light of their contribution to human health and plant defense systems, these phytochemicals, especially isoflavones, are desirable target traits in soybean breeding programs^48–53^. Isoflavones, synthesized predominantly in legumes, attract rhizobial bacteria, initiate nitrogen-fixing root nodule formation, exert antifungal activity, and serve as metabolic precursors for major phytoalexins^5,6,54,55^. CMS, a coumestan isoflavone, decreases the risk of breast cancer by binding selectively to ERβ^23,24,26^. Therefore, CMS is a promising phytoestrogen for use as a selective estrogen receptor modulator (SERM)^26^.

The extreme variability of isoflavone contents among different environments has hindered elucidation of the genetic basis of isoflavone biosynthesis^56–58^. Indeed, in this study, even though plant samples were prepared at the same location over 2 years in three biological replicates in each year, variations in CMS content were observed between 2016 and 2017, indicating that this trait is environmentally sensitive (Supplementary Fig. 1, Fig. 1A). Therefore, we only used leaf samples from genotypes with relatively consistent levels of CMS over both years. Moreover, to ensure that leaf tissues containing the highest levels of CMS were used for RNA-seq, the CMS content in Daewonkong was measured at two different time points at developmental stage R7 in each year of cultivation (Fig. 1B); CMS content dramatically increases after the reproductive stage and peaks at R7^42^. In 2016, CMS content increased about 3.5% as the leaves matured at R7, but in 2017 it increased about 41% only during R7. The Daewonkong leaf samples used for the second measurement in 2017, which had the highest CMS, were used for RNA-seq. The other genotype, SS0903-2B-21-1-2, exhibited consistently low CMS content in both cultivation years.

In total, we identified 4,046 DEGs between growth stage R7 leaf samples of Daewonkong and SS0903-2B-21-1-2. Out of eight DEGs in the functional co-expression network of the phenylpropanoid biosynthetic pathway (Fig. 3), six were up-regulated at least 8-fold in Daewonkong in comparison with SS0903-2B-21-1-2 (Fig. 2B), and five (CHS7, CHS8, CHR14, HIDH, and IFS2) in the downstream pathway are tightly connected with each other. Glyma.14G005700 (CHR14) is a candidate gene for an isoflavone QTL^59^, and Glyma.11G011500 (CHS8) and Glyma.13G173500 (IFS2) are differentially expressed under abiotic stress^60^. Moreover, the enzymes encoded by Glyma.01G228700 (CHS7), Glyma.11G011500 (CHS8), Glyma.13G173500 (IFS2), and Glyma14G005700 (CHR14) interact with each other to promote isoflavonoid synthesis^61^. According to our expression data and network analysis, along with the previously reported literature, seven of the eight DEGs (i.e., all but Glyma.16G149300) are likely to play key roles in the phenylpropanoid biosynthetic pathways with CMS as their final product (Supplementary Table 3).

The legume-specific isoflavonoid pathway includes several side pathways that overlap and compete with each other, and share daidzein as a central metabolite^62^. Among homologous genes involved in isoflavonoid biosynthesis with daidzein as the common precursor, four (Glyma.08G089500, Glyma.10G176700, Glyma.13G173600, and Glyma.16G149300) were connected in the co-expression network. Glyma.08G089500, Glyma.10G176700, and Glyma.13G173600 were minimally expressed in both genotypes, but Glyma.16G149300 was detected as a DEG. Glyma.16G149300 was down-regulated in Daewonkong, consistent with the higher level of CMS accumulation in this genotype (Fig. 3).

Other than the candidate genes that catalyze the NADPH oxidation reactions starting from daidzein, there are seven more candidate genes involved in the biosynthesis pathway of CMS. MYB domain protein 15 (Glyma.02G005600) and major latex protein (MLP)-like protein 423 (Glyma.17G030400) are annotated as involved in stress responses. Because the regulation of isoflavonoid metabolism is thought to occur primarily at the level of transcription, transcription factors (TFs) are promising candidates^63^. Multiple MYB TFs regulate the expression of structural genes involved in isoflavone biosynthesis under stressed conditions^64–66^. MLP-like protein modulates the production of metabolites under drought stress conditions, and overexpression of MLP leads to salt stress insensitivity^67,68^. This agrees well with reports that CMS increases drought stress through communication with mycorrhiza and CMS accumulates the most at R7 growth stage when soybean is dehydrated while maturation^33,42,69^. Thus, these two genes may induce accumulation of CMS under stress conditions.

For candidate DEGs unmapped to any GO term, Glyma.01G135200 and Glyma.13G285300 are annotated as cytochrome P450, an oxidoreductase, that is reported to be involved in isoflavonoid biosynthesis^70,71^. Glyma.11G004200, annotated as a member of the alpha/beta-hydrolase superfamily, may play a role in the hydrolysis reaction of CMS biosynthesis. 2-hydroxyisoflavanone dehydratase, which catalyzes a dehydration reaction yielding isoflavone from 2-hydroxyisoflavanone, is a member of the carboxyesterase family (Glyma.02G134000)^72^. To date, although no evidence for their direct roles in isoflavone biosynthesis has been reported, these genes could also affect the biosynthetic pathway of CMS in as-yet-undiscovered ways (Fig. 5).

The functions of the candidate genes remain to be experimentally verified. The biosynthetic pathways responsible for production of anthocyanins, another class of soybean flavonoid metabolites, have been well studied due to the importance of these compounds for human health and the cosmetic industry. Anthocyanin content in soybean seeds is determined by six loci, and all genes corresponding to these loci have been isolated^73–78^. In addition, the key enzymes could be characterized by forward genetic approaches because anthocyanin levels can be easily distinguished based on the color of the seed coat or flower. However, because variations in CMS content do not cause any visible phenotypic variation, identification of the candidate genes involved in CMS biosynthesis is essential for study of the biosynthetic pathway using reverse genetic approaches. Knockout or down-regulation of each candidate gene, or multiple genes at the same time, would enable characterization of their effects on CMS accumulation. *In vitro* conversion of intermediate products with candidate enzymes might also be a good approach to characterizing the role of each enzyme in CMS biosynthesis.

In summary, to determine the genetic basis of CMS accumulation, we sequenced RNA samples from two genotypes, Daewonkong and SS0903-2B-21-1-2, which had consistently high and low CMS content, respectively. Using a co-expression network database and key DEGs identified in the iso/flavonoid biosynthetic pathway, we identified genes that might play important roles in CMS accumulation. Our results provide a valuable resource to help elucidate CMS biosynthesis in soybean and develop soybean cultivars with desired CMS contents, with the aim of improving plant defense and human health. Future research will focus on functional validation of the identified genes and complete characterization of the CMS biosynthetic pathway.

## Materials and Methods

### Plant materials for HPLC analysis

For CMS measurements, 31 soybean genotypes were planted at the Seoul National University Experimental Farm in Suwon, South Korea (37.3 °N, 127.0 °E), with three replications per cultivar, in 2016 (Supplementary Fig. 1). Average temperature and duration of sunshine in Suwon from May to October of 2016 were 16.1–28.0 °C and 1,293.6 hr, respectively. In 2017, eight genotypes, including four with high CMS and four with low CMS in 2016, were planted again in three replicates (Fig. 1A). Average temperature and duration of sunshine in Suwon from May to October of 2017 were 16.4–26.9 °C and 1,334.4 hr, respectively.

Soybean leaves collected at growth stage R7 were dried and ground to a fine powder. Each sample (3 g) was stirred in 45 mL of 80% ethanol for 1 day at room temperature, and then the mixture was filtered through a 0.45 μm GHP membrane filter (Acrodisc 13 mm syringe filter; Pall Corporation, Port Washington, NY, USA). Quantitative analysis of CMS was performed on a Mightysil RP-18 GP reversed phase column (5 μm, 4.6 × 250 mm) (Kanto Chemical Co., Tokyo, Japan) at sub-ambient temperature, using a 40 min linear gradient of 0.1% glacial acetic acid in water (solvent A) and 0.1% glacial acetic acid in acetonitrile (solvent B). The linear gradient program was as follows: 0–5 min, 31% B; 5–25 min, 31–35% B; 25–30 min, 35–80% B; 30–35 min, 80% B; 35–40, min back to 31% B. The solvent flow rate was 1.0 mL min^–1^, and the injection volume was 10 μL. UV absorption was measured at 342 nm to detect CMS (the standard purchased from Sigma-Aldrich Co., St Louis, MO, USA).

### RNA-seq

Total RNA was extracted from growth stage R7 leaf samples of Daewonkong and SS903-B2-21-1-2 in 2017 using Ribospin^™^ Plant (GeneAll, Seoul, Korea). Three cDNA libraries per genotype were constructed using the TruSeq^®^ RNA Sample Prep Kit v2 (Illumina Inc., CA, USA). The quality and quantity of samples used for sequencing were checked using a 2100 Bioanalyzer (Agilent Technologies). RNA samples were sequenced using the TruSeq SBS kit v3 on the Illumina HiSeq. 4000 platform. The raw RNA reads of Daewonkong (SRR6756974, SRR6756973, and SRR6756972) and SS903-B2-21-1-2 (SRR6756971, SRR6756976, and SRR6756975) have been deposited in the National Center for Biotechnology Information (NCBI) Sequence Read Archive (Supplementary Table 1).

### DEGs and enrichment analysis

Fragments Per Kilobase of transcript per Million mapped reads (FPKM) values were calculated by mapping raw RNA reads for 56,044 genes to the *G. max* reference genome annotation data (Gmax_275_Wm82.a2.v1.gene.gff3) using the Tuxedo software suite^79^. DEGs were defined as genes with a log_2_ fold change (FC) ≥ 2 between two samples in pairwise comparisons for three replications with *p*-value < 0.05 (Fig. 2A). FPKM values < 1 were converted to 1 for the purpose of calculating FC. Sets of DEGs with log_2_FC ≥ 2, 3, 4, or 5 were used for Gene Ontology (GO) enrichment analysis using the Singular Enrichment Analysis (SEA) tool, available at agriGO (http://bioinfo.cau.edu.cn/agriGO/), and KEGG (Kyoto Encyclopedia of Genes and Genomes) ontology (http://www.genome.jp/kegg/tool/map_pathway1.html) (*p* < 0.05) (Supplementary Fig. 2). The *G. max* reference annotation (Wm82.a2.v1) was used as a background reference for enrichment analysis.

### Candidate gene prediction

New candidate genes in the phenylpropanoid biosynthetic pathway were identified using the soybean co-expression network consisting of 1,940,284 co-functional links between 40,812 soybean genes constructed based on 734 microarrays and 290 RNA-seq data from soybean, available at http://www.inetbio.org/soynet/ ^44^. First, new candidate genes in the pathway were identified by searching genes closely connected to the 41 known genes in the pathway as guide genes in the co-expression network (“guide prediction”). Second, the seven DEGs among the 41 genes in the pathway were used as guide genes for the same function. The genes were prioritized according to the sum of their log-likelihood scores, and highly ranked genes were considered good candidates for new members of the pathway^44^. Third, DEGs was used to identify new candidate genes through subnetworks consisting of a central hub and their neighbors (“hub prediction”). If significant overlap was observed between DEGs and neighbor genes of a subnetwork, the central hub of the subnetwork was identified as a candidate gene. Candidate genes were prioritized according to their *p*-values.

### qRT-PCR validation of DEGs

Gene-specific primers for qRT-PCR analysis were designed based on the nucleotide sequences of selected DEGs using Primer3 (http://primer3plus.com/) (Supplementary Table 2). cDNA was synthesized using an iScript^™^ cDNA Synthesis Kit (Cat. 170-8891; Bio-Rad, Hercules, CA, USA). qRT-PCR was conducted using an iQ^™^ SYBR Green Supermix kit (Cat. 170-8882; Bio-Rad) on a LightCycler^®^ 480 (Roche Diagnostics, Laval, QC, Canada). *Actin* was used for normalization of target gene expression, and each sample was analyzed in triplicate. Relative gene expression was analyzed based on the reference gene as previously described^80^. Student’s t-test was performed to determine whether differences were statistically significant (*p* < 0.05).

## Supplementary information

Supplementary data

## Data Availability

The raw RNA sequencing reads were deposited at NCBI SRA.

## References

[1] Wink M (2015). Modes of action of herbal medicines and plant secondary metabolites. Medicines.

[2] Kabera JN, Semana E, Mussa AR, He X (2014). Plant secondary metabolites: biosynthesis, classification, function and pharmacological properties. J Pharm Pharmacol.

[3] Lee CH (2005). Relative antioxidant activity of soybean isoflavones and their glycosides. Food Chem..

[4] Messina MJ, Persky V, Setchell KD, Barnes S (1994). Soy intake and cancer risk: a review of the *in vitro* and *in vivo* data. Nutr. Cancer.

[5] Park S, Lee W, Park Y, Ahn J (2006). Effects of nitrogen source and bacterial elicitor on isoflavone accumulation in root cultures of *Albizzia kalkora* (Roxb.) Prain. J. Integr. Plant Biol..

[6] Dixon RA, Paiva NL (1995). Stress-induced phenylpropanoid metabolism. Plant Cell.

[7] Ososki AL, Kennelly EJ (2003). Phytoestrogens: a review of the present state of research. Phytother. Res..

[8] Albertazzi P, Purdie DW (2002). The nature and utility of the phytoestrogens: a review of the evidence. Maturitas.

[9] Perna S (2016). Multidimensional Effects of soy isoflavone by food or supplements in menopause women: a systematic review and bibliometric analysis. Nat. Prod. Commun..

[10] Steinberg FM, Guthrie NL, Villablanca AC, Kumar K, Murray MJ (2003). Soy protein with isoflavones has favorable effects on endothelial function that are independent of lipid and antioxidant effects in healthy postmenopausal women. Am. J. Clin. Nutr..

[11] Patel RP (2001). Antioxidant mechanisms of isoflavones in lipid systems: paradoxical effects of peroxyl radical scavenging. Free Radic. Biol. Med..

[12] Dixon RA (2004). Phytoestrogens. Annu. Rev. Plant Biol..

[13] Cornwell T, Cohick W, Raskin I (2004). Dietary phytoestrogens and health. Phytochemistry.

[14] Hall G, Phillips TJ (2005). Estrogen and skin: the effects of estrogen, menopause, and hormone replacement therapy on the skin. J. Am. Acad. Dermatol..

[15] Macgregor JI, Jordan VC (1998). Basic guide to the mechanisms of antiestrogen action. Pharmacol. Rev..

[16] Cummings SR (2002). Serum estradiol level and risk of breast cancer during treatment with raloxifene. Jama.

[17] Writing group for the women’s health initiative investigators (2002). Risks and benefits of estrogen plus progestin in healthy postmenopausal women: principal results from the Women’s Health Initiative randomized controlled trial. Jama.

[18] Green S (1986). Cloning of the human oestrogen receptor cDNA. J. Steroid Biochem..

[19] Levenson A, Jordan V (1999). Selective oestrogen receptor modulation: molecular pharmacology for the millennium. Eur. J. Cancer.

[20] Paruthiyil S (2004). Estrogen receptor β inhibits human breast cancer cell proliferation and tumor formation by causing a G2 cell cycle arrest. Cancer Res..

[21] Yuk HJ (2011). The most abundant polyphenol of soy leaves, coumestrol, displays potent α-glucosidase inhibitory activity. Food Chem..

[22] Collins BM, McLachlan JA, Arnold SF (1997). The estrogenic and antiestrogenic activities of phytochemicals with the human estrogen receptor expressed in yeast. Steroids.

[23] Martin PM, Horwitz KB, Ryan DS, Mcguire WL (1978). Phytoestrogen interaction with estrogen receptors in human breast cancer cells. Endocrinology.

[24] Stahl S, Chun T-Y, Gray WG (1998). Phytoestrogens act as estrogen agonists in an estrogen-responsive pituitary cell line. Toxicol. Appl. Pharmacol..

[25] Bickoff E (1957). Coumestrol, a new estrogen isolated from forage crops. Sci. Wash..

[26] Choi SY (2008). Estrogenic activities of isoflavones and flavones and their structure-activity relationships. Planta Med..

[27] Hedelin M (2008). Dietary phytoestrogens are not associated with risk of overall breast cancer but diets rich in coumestrol are inversely associated with risk of estrogen receptor and progesterone receptor negative breast tumors in Swedish women. J. Nutr..

[28] Park G (2015). Flt3 is a target of coumestrol in protecting against UVB-induced skin photoaging. Biochem. Pharmacol..

[29] Hwang JA (2017). Coumestrol down-regulates melanin production in melan-a murine melanocytes through degradation of tyrosinase. Biol. Pharm. Bull..

[30] Boué SM, Carter CH, Ehrlich KC, Cleveland TE (2000). Induction of the soybean phytoalexins coumestrol and glyceollin by. Aspergillus. J. Agric. Food Chem..

[31] Lee JH (2006). LDL-antioxidant pterocarpans from roots of *Glycine max* (L.) Merr. J. Agric. Food Chem..

[32] Xie Z-P (1995). Rhizobial nodulation factors stimulate mycorrhizal colonization of nodulating and nonnodulating soybeans. Plant Physiol..

[33] Tripathi P (2016). A toolbox of genes, proteins, metabolites and promoters for improving drought tolerance in soybean includes the metabolite coumestrol and stomatal development genes. BMC Genomics.

[34] Samanta, A., Das, G. & Das, S. K. Roles of flavonoids in plants. *carbon* **100** (2011).

[35] Lee H-I, Lee J-H, Park K-H, Sangurdekar D, Chang W-S (2012). Effect of soybean coumestrol on *Bradyrhizobium japonicum* nodulation ability, biofilm formation, and transcriptional profile. Appl. Environ. Microbiol..

[36] Dewick P, Barz W, Grisebach H (1970). Biosynthesis of coumestrol in *Phaseolus aureus*. Phytochemistry.

[37] Berlin J, Dewick P, Barz W, Grisebach H (1972). Biosynthesis of coumestrol in *Phaseolus aureus*. Phytochemistry.

[38] Eldridge AC, Kwolek WF (1983). Soybean isoflavones: effect of environment and variety on composition. J. Agric. Food Chem..

[39] Zeng G (2009). Identification of QTL underlying isoflavone contents in soybean seeds among multiple environments. Theor. Appl. Genet..

[40] Kassem M (2006). An updated ‘Essex’by ‘Forrest’linkage map and first composite interval map of QTL underlying six soybean traits. Theor. Appl. Genet..

[41] Gutierrez-Gonzalez JJ (2009). Genetic control of soybean seed isoflavone content: importance of statistical model and epistasis in complex traits. Theor. Appl. Genet..

[42] Yun D-Y (2016). Distinctive metabolism of flavonoid between cultivated and semiwild soybean unveiled through metabolomics approach. J. Agric. Food Chem..

[43] Schmutz J (2010). Genome sequence of the palaeopolyploid soybean. Nature.

[44] Kim E, Hwang S, Lee I (2017). SoyNet: a database of co-functional networks for soybean *Glycine max*. Nucleic Acids Res..

[45] Stevens, L. & Price, N. C. Fundamentals of enzymology: the cell and molecular biology of catalytic proteins (1999).

[46] Hanukoglu I (2015). Proteopedia: Rossmann fold: A beta‐alpha‐beta fold at dinucleotide binding sites. Biochem. Mol. Biol. Educ..

[47] Stammers DK (2001). The structure of the negative transcriptional regulator NmrA reveals a structural superfamily which includes the short‐chain dehydrogenase/reductases. EMBO J..

[48] Gou L (2016). Multigene synergism increases the isoflavone and proanthocyanidin contents of *Medicago truncatula*. Plant Biotechnol. J..

[49] Li P (2016). Metabolic engineering of proanthocyanidin production by repressing the isoflavone pathways and redirecting anthocyanidin precursor flux in legume. Plant Biotechnol. J..

[50] Jhan J-K (2016). Anthocyanin contents in the seed coat of black soya bean and their anti-human tyrosinase activity and antioxidative activity. Int. J. Cosmet. Sci..

[51] Ha J (2017). Transcriptomic variation in proanthocyanidin biosynthesis pathway genes in soybean (*Glycine* spp.). J. Sci. Food Agric..

[52] Dastmalchi M, Chapman P, Yu J, Austin RS, Dhaubhadel S (2017). Transcriptomic evidence for the control of soybean root isoflavonoid content by regulation of overlapping phenylpropanoid pathways. BMC Genomics.

[53] Zabala G (2006). Transcriptome changes in the phenylpropanoid pathway of *Glycine max* in response to Pseudomonas syringaeinfection. BMC Plant Biol..

[54] Pueppke SG (1996). The genetic and biochemical basis for nodulation of legumes by rhizobia. Crit. Rev. Biotechnol..

[55] Rivera-Vargas LI, Schmitthenner AF, Graham TL (1993). Soybean flavonoid effects on and metabolism by *Phytophthora sojae*. Phytochemistry.

[56] Dhaubhadel S, McGarvey BD, Williams R, Gijzen M (2003). Isoflavonoid biosynthesis and accumulation in developing soybean seeds. Plant Mol. Biol..

[57] Bennett JO, Yu O, Heatherly LG, Krishnan HB (2004). Accumulation of genistein and daidzein, soybean isoflavones implicated in promoting human health, is significantly elevated by irrigation. J. Agric. Food Chem..

[58] Lozovaya VV (2005). Effect of temperature and soil moisture status during seed development on soybean seed isoflavone concentration and composition. Crop Sci..

[59] Pei, R. *et al*. Identification of novel QTL associated with soybean isoflavone content. *Crop J* (2018).

[60] Zeng W (2017). Comparative transcriptome analysis of soybean response to bean pyralid larvae. BMC Genomics.

[61] Dastmalchi M, Bernards MA, Dhaubhadel S (2016). Twin anchors of the soybean isoflavonoid metabolon: evidence for tethering of the complex to the endoplasmic reticulum by IFS and C4H. Plant J..

[62] Dixon RA, Pasinetti GM (2010). Flavonoids and isoflavonoids: from plant biology to agriculture and neuroscience. Plant Physiol..

[63] Hichri I (2011). Recent advances in the transcriptional regulation of the flavonoid biosynthetic pathway. J. Exp. Bot..

[64] Shelton D (2012). Transcription factors of Lotus: regulation of isoflavonoid biosynthesis requires coordinated changes in transcription factor activity. Plant Physiol..

[65] Yi J (2010). A single‐repeat MYB transcription factor, GmMYB176, regulates CHS8 gene expression and affects isoflavonoid biosynthesis in soybean. Plant J..

[66] Du H (2012). Genome-wide analysis of the MYB transcription factor superfamily in soybean. BMC Plant Biol..

[67] Chen J-Y, Dai X-F (2010). Cloning and characterization of the *Gossypium hirsutum* major latex protein gene and functional analysis in *Arabidopsis thaliana*. Planta.

[68] Wang Y (2016). Major latex protein-like protein 43 (MLP43) functions as a positive regulator during abscisic acid responses and confers drought tolerance in *Arabidopsis thaliana*. J. Exp. Bot..

[69] Fehr WR, Caviness CE, Burmood D, Pennington J (1971). Stage of development descriptions for soybeans, *Glycine Ma*x (L.)Merrill 1. Crop Sci..

[70] Schuler MA, Werck-Reichhart D (2003). Functional genomics of P450s. Annu. Rev. Plant Biol..

[71] Harvey PJ (2002). Phytoremediation of polyaromatic hydrocarbons, anilines and phenols. Environ. Sci. Pollut. Res..

[72] Akashi T, Aoki T, Ayabe S (2005). Molecular and biochemical characterization of 2-hydroxyisoflavanone dehydratase. Involvement of carboxylesterase-like proteins in leguminous isoflavone biosynthesis. Plant Physiol..

[73] Gillman JD, Tetlow A, Lee J-D, Shannon JG, Bilyeu K (2011). Loss-of-function mutations affecting a specific *Glycine max* R2R3 MYB transcription factor result in brown hilum and brown seed coats. BMC Plant Biol..

[74] Tuteja JH, Zabala G, Varala K, Hudson M, Vodkin LO (2009). Endogenous, tissue-specific short interfering RNAs silence the chalcone synthase gene family in *Glycine max* seed coats. Plant Cell.

[75] Yang K (2010). Genetic analysis of genes controlling natural variation of seed coat and flower colors in soybean. J. Hered..

[76] Zabala G, Vodkin L (2003). Cloning of the pleiotropic T locus in soybean and two recessive alleles that differentially affect structure and expression of the encoded flavonoid 3′ hydroxylase. Genetics.

[77] Zabala G, Vodkin LO (2005). The wp mutation of *Glycine max* carries a gene-fragment-rich transposon of the CACTA superfamily. Plant Cell.

[78] Zabala, G. & Vodkin, L. O. Rearrangement resulting in small tandem repeats in the F3′5′H gene of white flower genotypes is associated with the soybean W1 locus. *Crop Sci* (2007).

[79] Trapnell C (2012). Differential gene and transcript expression analysis of RNA-seq experiments with TopHat and Cufflinks. Nat. Protoc..

[80] Livak KJ, Schmittgen TD (2001). Analysis of relative gene expression data using real-time quantitative PCR and the 2−ΔΔCT method. Methods.

