# Supplementary material for: Comprehensive RNA sequencing and co-expression network analysis to complete the biosynthetic pathway of coumestrol, a phytoestrogen
Source: Sci Rep. 2019 Feb 13;9:1934. doi: 10.1038/s41598-018-38219-6 (PMC6374408; doi:10.1038/s41598-018-38219-6)
Supplement: Supplementary file 1 — Supplementary data [file 41598_2018_38219_MOESM1_ESM.docx]

**Comprehensive RNA sequencing and co-expression network analysis to complete the biosynthetic pathway of coumestrol, a phytoestrogen**

Jungmin Ha^1,2^, Young-Gyu Kang^3^, Taeyoung Lee^1^, Myoyeon Kim^3^, Min Young Yoon^1,2^, Eunsoo Lee^1^, Xuefei Yang^1^, Donghyun Kim^3^, Yong-Jin Kim^3^, Tae Ryong Lee^3^, Moon Young Kim^1,2^, Suk-Ha Lee^1,2*^

^1^Department of Plant Science and Research Institute of Agriculture and Life Sciences, Seoul National University, Seoul, Republic of Korea

^2^Plant Genomics and Breeding Institute, Seoul National University, Seoul, Republic of Korea

^3^Basic Research & Innovation Division, R&D Center, AmorePacific Corporation, Republic of Korea

*Correspondence: Suk-Ha Lee

E-mail address: sukhalee@snu.ac.kr

Address: Crop Genomics Lab. Rm. 4105 Bldg. 200 CALS, Seoul National University, 1 Gwanak-ro, Gwanak-gu, Seoul 08826, Republic of Korea

Tel: +8228804545

Fax: +8228774550

Supplementary Figure 1. CMS contents in soybean leaf samples at R7 growth stage of 31 genotypes.

For CMS measurement, 31 Soybean genotypes were planted at the Seoul National University Experimental Farm in Suwon, South Korea with three replications in 2016. Four genotypes with high CMS contents indicated by red bars (1: Geomjeongsaeol, 3: Chamame, 16: SG-257, 19: Daewonkong) and four genotypes with low CMS contents indicated by blue bars (13: Haepum, 14: SS0903-2B-21-1-2, 15: SS0905-2B-179-1-1, 18: Sinhwa) were selected for replicated experiment in 2017. Error bar indicates standard deviation. (1: Geomjeongsaeol, 2: SS0911-2B-35-1-2-3-4, 3: Chamame, 4: Napjakong, 5: TS245, 6: SS0404-T5-63, 7: SS0408-T5-99, 8: PSN662, 9: SS0903-2B-27-2-1-1-3, 10: SS0905-2SSD-179-1-1-1-1, 11: Hwangkeumkong, 12: IT182932, 13: Haepum, 14: SS0903-2B-21-1-2, 15: SS0905-2B-179-1-1, 16: SG-257, 17: Jinpung, 18: Sinhwa, 19: Daewonkong, 20: Uram, 21: Jangwonkong, 22: SS0910-2B-14-3-3-3-2, 23: SS0910-2B-30-2-4-4-1, 24: SS0905-2SSD-184-1-4-1-2, 25: SS0905-2SSD-522-1-4-1-4, 26: SS0908-4SSD-115-1-4, 27: SS09010-25-1-4-4, 28: SS0908-6SSD-136, 29: SS0905-5SSD-544, 30: SS0908-6SSD-104, 31: Saedanbaek).

| Sample | Tissue type | Growth stage | Library type | Read count | Total base | Read count properly mapped | ^*^SRA |
| --- | --- | --- | --- | --- | --- | --- | --- |
| Daewonkong-1 | leaf | R7 | Paired | 81,652,916 | 8,246,944,516 | 58,626,342 | SRR6756974 |
| Daewonkong-2 | leaf | R7 | Paired | 74,090,454 | 7,483,135,854 | 51,310,894 | SRR6756973 |
| Daewonkong-3 | leaf | R7 | Paired | 79,633,944 | 8,043,028,344 | 54,516,626 | SRR6756972 |
| SS903-1 | leaf | R7 | Paired | 82,433,370 | 8,325,770,370 | 64,121,720 | SRR6756971 |
| SS903-2 | leaf | R7 | Paired | 67,171,614 | 6,784,333,014 | 50,640,998 | SRR6756976 |
| SS903-3 | leaf | R7 | Paired | 95,152,744 | 9,610,427,144 | 68,594,808 | SRR6756975 |

Supplementary Table 1. General statistics of RNA raw reads sequenced by Hiseq4000 platform.

The raw reads were deposited in NCBI SRA.

^*^SRA: Sequence read archive

Supplementary Figure 2. KO enrichment analysis.

The ratio of genes identified in the KEGG pathways related to secondary metabolites biosynthesis are shown. The Y-axis indicates the names of KEGG pathways; the X axis indicates the percentage of genes in each pathway.

Supplementary Table 2. List of primer sets for qRT-PCR.

| Gene ID | Forward sequence (5’-3’) | Reverse sequence (5’-3’) | Product size (bp) | Tm (℃) |
| --- | --- | --- | --- | --- |
| Glyma.01G135200 | TCATGAACGTGATGCTTTCC | GCTTGCTTCAGTTCCTGCTT | 115 | 55 |
| Glyma.02G005600 | CTTGGACCCCAGAGGAAGAT | ACCTTAGCCTGCAGCTCTTG | 123 | 55 |
| Glyma.02G134000 | TTTCGGGAAAGTGTTTTTCG | CGCTCCAACAGAAATTCAGA | 129 | 55 |
| Glyma.02G307300 | GGTCACCGAAAATCATCCTC | GAGAACTTCCCTGGCTGAGA | 126 | 55 |
| Glyma.03G147700 | CCGAACAGGACTCGAAACTT | AAATCTCCATCGGTGAATGC | 112 | 55 |
| Glyma.09G269500 | GTCACTGGAGGCACAGGATT | GCCTGGTAGGTTTGTGAGGA | 135 | 55 |
| Glyma.11G004200 | GCCAACAACACCAACAACAC | ACGCAAAAGCAAGTTGTGTG | 120 | 55 |
| Glyma.11G070500 | TCCTGAAACCAAGGAAGAGC | CCAAATGCACAGATTACGACA | 132 | 55 |
| Glyma.13G284900 | GGGTTTGATTTGCGGAGATA | CTGAGAGGTGGCCAAATGTT | 98 | 55 |
| Glyma.13G285300 | ATGAGCGTGTTGCTTTCCTT | AATGCTTGCCTCAGTTCCTG | 114 | 55 |
| Glyma.17G030400 | GCCCTGGAACAATCAAGAAG | ATCCCAAGTTTGCCTCATCA | 98 | 55 |
| Glyma.18G220600 | CAGGATTCCTTGGTTCATGG | TGCACCAGGTAGGTTTGTGA | 125 | 55 |
| Glyma.18G285800 | TGGGTCACTGATAACCATGC | GGCCAGTGGATCAGAAAGAG | 101 | 55 |
| Glyma.19G030500 | ATGGATGAACTTCGGTGGAG | TATCCAACGGTGTCAACTCG | 113 | 55 |
| Actin | CGGTGGTTCTATCTTGGCATC | GTCTTTCGCTTCAATAACCCTA | 142 | 55 |


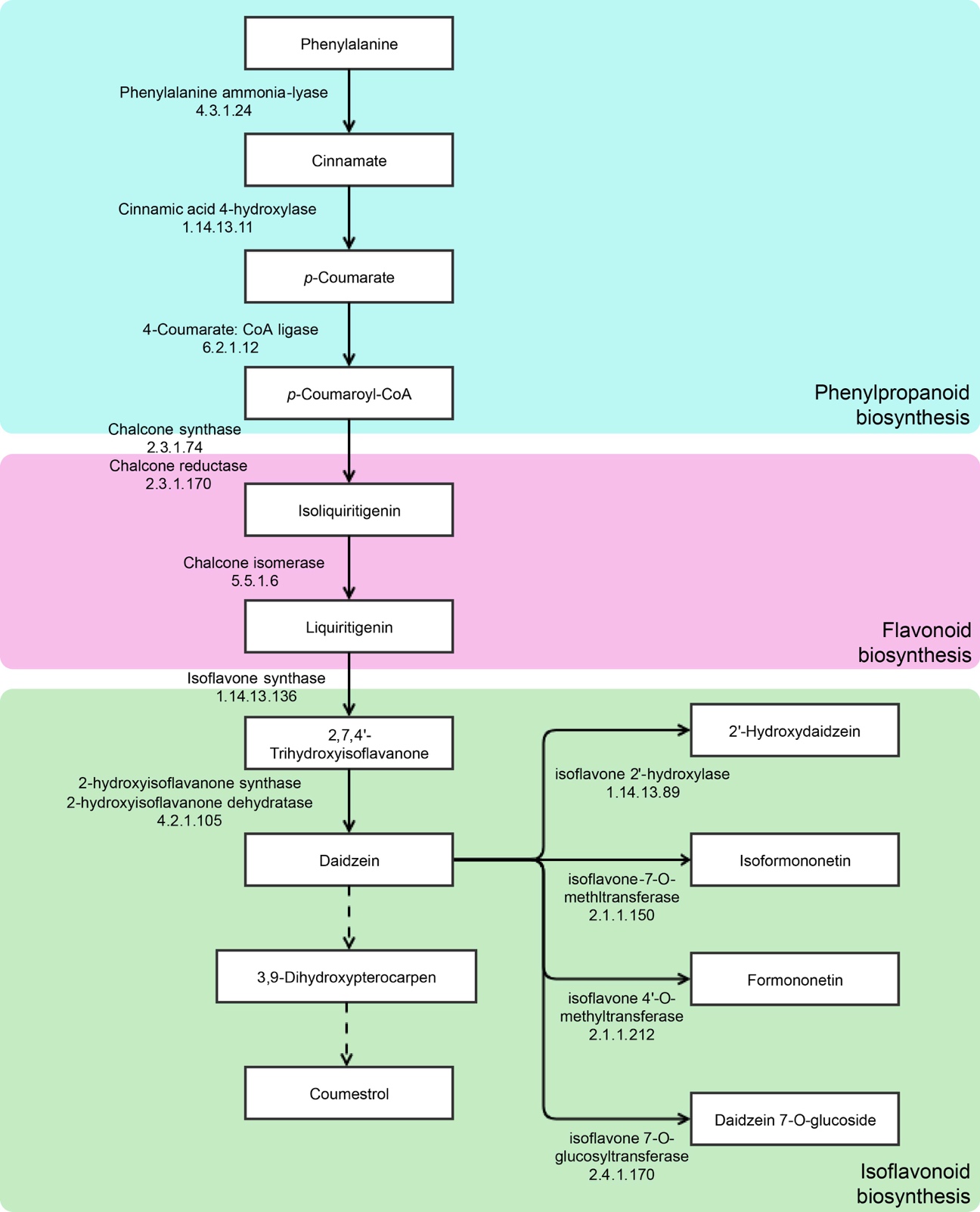


Supplementary Figure 3. Biosynthetic pathways of CMS.

From phenylalanine to CMS, intermediate byproducts are indicated in the boxes. Key enzymes and EC numbers for each pathway are indicated next to the arrows. Dotted lines represent unknown pathways.

Supplementary Table 3. List of DEGs in the co-expression network.

| EC number | Gene name | Gene symbol | At homolog | Description |
| --- | --- | --- | --- | --- |
| 2.3.1.74 | Glyma.01G228700 | CHS7 | AT5G13930.1 | Chalcone and stilbene synthase family protein |
| 4.2.1.105 | Glyma.01G239600 | HIDH | AT1G47480.1 | alpha/beta-Hydrolases superfamily protein |
| 6.2.1.12 | Glyma.11G010500 | 4CL13 | AT1G65060.1 | 4-coumarate:CoA ligase 3 |
| 2.3.1.74 | Glyma.11G011500 | CHS8 | AT5G13930.1 | Chalcone and stilbene synthase family protein |
| 1.14.13.136 | Glyma.13G173500 | IFS2 | AT5G06900.1 | cytochrome P450, family 93, subfamily D, polypeptide 1 |
| 2.3.1.170 | Glyma.14G005700 | CHR14 | AT1G59960.1 | NAD(P)-linked oxidoreductase superfamily protein |
| 1.14.13.89 | Glyma.16G149300 | LOC100811727 | AT4G37330.1 | cytochrome P450, family 81, subfamily D, polypeptide 4 |
| 6.2.1.12 | Glyma.17G064400 | LOC100779668 | AT3G21240.1 | 4-coumarate:CoA ligase 2 |


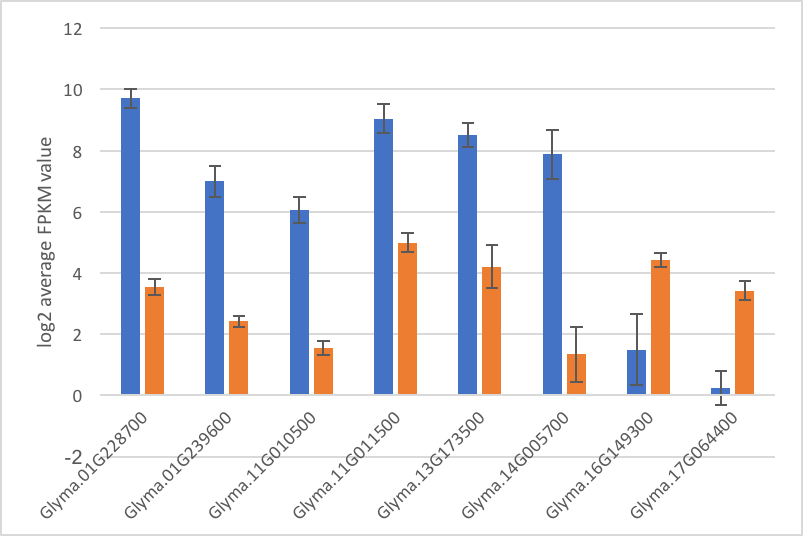


Supplementary Figure 4. Expression levels of seven DEGs in the co-expression network.

The gene expressions in Daewonkong and SS0903-B2-21-1-2 were indicated by blue and orange bars, respectively. The average FPKM values were converted to logarithmic space by taking log 2. (Glyma.01G228700, 2.3.1.74, Chalcone and stilbene synthase family protein; Glyma.01G239600, 4.2.1.105, alpha/beta-Hydrolases superfamily protein; Glyma.11G010500, 6.2.1.12, 4-coumarate:CoA ligase 3; Glyma.11G011500, 2.3.1.74, Chalcone and stilbene synthase family protein; Glyma.13G173500, 1.14.13.136, cytochrome P450, family 93, subfamily D, polypeptide 1; Glyma.14G005700, 2.3.1.170, NAD(P)-linked oxidoreductase superfamily protein; Glyma.16G149300, 1.14.13.89, cytochrome P450, family 81, subfamily D, polypeptide 4; Glyma.17G064400, 6.2.1.12, 4-coumarate:CoA ligase 2).

Supplementary Table 4. List of candidate genes predicted by three approaches.

GO terms for each gene from three databases are listed. DEGs are indicated in bold.

|  | rank | predicted genes | paralogs | AgriGO-BP | At GO | Uniprot GO |
| --- | --- | --- | --- | --- | --- | --- |
| Guide prediction using 41 genes in network | 1 | Glyma.15G136900 | Glyma.09G031900 | na | na | na |
|  | 2 | Glyma.09G031900 | Glyma.15G136900 | na | na | na |
|  | 3 | Glyma.04G105000 | Glyma.06G106000 | small GTPase mediated signal transduction;ferrous iron transport | na | GTP catabolic process;tRNA processing;tRNA modification |
|  | 4 | Glyma.06G106000 | Glyma.04G105000 | small GTPase mediated signal transduction;ferrous iron transport | na | GTP catabolic process;tRNA processing;tRNA modification |
|  | 5 | Glyma.03G250800 | Glyma.10G166000 | biosynthetic process;cellular amino acid metabolic process | threonine catabolic process | cellular amino acid metabolic process |
|  | 6 | Glyma.09G148100 | no_paralog | na | na | na |
|  | 7 | Glyma.16G199200 | no_paralog | na | na | na |
|  | 8 | Glyma.20G223200 | Glyma.03G250800 | biosynthetic process;cellular amino acid metabolic process | threonine catabolic process | cellular amino acid metabolic process |
|  | 9 | Glyma.10G166000 | Glyma.03G250800 | biosynthetic process;cellular amino acid metabolic process | threonine catabolic process | cellular amino acid metabolic process;threonine catabolic process |
|  | 10 | **Glyma.13G284900** | Glyma.06G263400 | na | na | na |
|  | 11 | **Glyma.11G004200** | Glyma.01G239300 | na | na | na |
|  | 12 | Glyma.12G216300 | Glyma.06G263400 | na | na | na |
|  | 13 | Glyma.06G263400 | Glyma.12G139000 | na | na | na |
|  | 14 | Glyma.12G139000 | Glyma.06G263400 | na | na | na |
|  | 15 | Glyma.12G216400 | no_paralog | na | na | cellular response to water deprivation |
|  | 16 | Glyma.16G103900 | Glyma.09G211500 | cellular metabolic process;steroid biosynthetic process | lignan biosynthetic process | na |
|  | 17 | Glyma.06G202300 | Glyma.05G021800 | na | flavonoid biosynthetic process | na |
|  | 18 | Glyma.19G121600 | Glyma.03G004300 | na | na | na |
|  | 19 | **Glyma.18G220600** | no_paralog | cellular metabolic process;steroid biosynthetic process | brassinosteroid metabolic process; flavonoid biosynthetic process; regulation of brassinosteroid biosynthetic process | cellular metabolic process |
|  | 20 | Glyma.04G107500 | Glyma.01G106000 | na | toxin catabolic process | na |
| Guide prediction using 7 DEGs | 1 | Glyma.16G103900 | Glyma.09G211500 | cellular metabolic process;steroid biosynthetic process | lignan biosynthetic process | na |
|  | 2 | **Glyma.11G004200** | Glyma.01G239300 | na | na | na |
|  | 3 | Glyma.20G241500 | Glyma.10G292200 | na | na | flavonoid biosynthetic process |
|  | 4 | **Glyma.02G307300** | **Glyma.14G005700** | na | na | flavonoid biosynthetic process |
|  | 5 | Glyma.19G121600 | Glyma.03G004300 | na | na | na |
|  | 6 | **Glyma.18G220600** | no_paralog | cellular metabolic process;steroid biosynthetic process | brassinosteroid metabolic process; flavonoid biosynthetic process; regulation of brassinosteroid biosynthetic process | cellular metabolic process |
|  | 7 | Glyma.09G040500 | **Glyma.17G030400** | defense response;response to biotic stimulus | na | defense response;response to biotic stimulus |
|  | 8 | **Glyma.18G285800** | no_paralog | na | na | na |
|  | 9 | Glyma.19G182300 | Glyma.03G181600 | biosynthetic process | defense response; drought recovery; lignin catabolic process; phenylpropanoid biosynthetic process; pollen development; response to UV-B; response to wounding; salicylic acid catabolic process | biosynthetic process;L-phenylalanine catabolic process;phenylpropanoid metabolic process |
|  | 10 | **Glyma.11G070500** | no_paralog | steroid biosynthetic process | na | na |
|  | 11 | Glyma.02G309300 | no_paralog | biosynthetic process | na | na |
|  | 12 | **Glyma.13G285300** | no_paralog | na | na | na |
|  | 13 | Glyma.08G109500 | no_paralog | fatty acid biosynthetic process | na | biosynthetic process |
|  | 14 | Glyma.20G209700 | **Glyma.02G005600** | na | na | response to salt stress;response to salicylic acid;response to jasmonic acid;response to gibberellin |
|  | 15 | **Glyma.19G030500** | Glyma.08G247100 | na | na | na |
|  | 16 | **Glyma.17G030400** | Glyma.09G040500 | defense response;response to biotic stimulus | na | defense response;response to biotic stimulus |
|  | 17 | **Glyma.09G269500** | no_paralog | cellular metabolic process;steroid biosynthetic process | brassinosteroid metabolic process; flavonoid biosynthetic process; regulation of brassinosteroid biosynthetic process | cellular metabolic process |
|  | 18 | Glyma.12G019900 | no_paralog | biosynthetic process;cellular metabolic process;steroid biosynthetic process | na | na |
|  | 19 | **Glyma.02G134000** | no_paralog | na | na | na |
|  | 20 | Glyma.04G230400 | no_paralog | na | na | na |
| Hub prediction using 7 DEGs | 1 | Glyma.02G309300 |  | biosynthetic process | na | na |
|  | 2 | Glyma.19G182300 | Glyma.20G180800 | biosynthetic process | defense response; drought recovery; lignin catabolic process; phenylpropanoid biosynthetic process; pollen development; response to UV-B; response to wounding; salicylic acid catabolic process | biosynthetic process;L-phenylalanine catabolic process;phenylpropanoid metabolic process |
|  | 3 | **Glyma.03G147700** |  | na | na | na |
|  | 4 | **Glyma.11G070500** |  | steroid biosynthetic process | na | na |
|  | 5 | Glyma.08G109500 |  | fatty acid biosynthetic process | na | biosynthetic process |
|  | 6 | Glyma.09G040500 | **Glyma.17G030400** | defense response;response to biotic stimulus | na | defense response;response to biotic stimulus |
|  | 7 | Glyma.20G241500 |  | na | na | flavonoid biosynthetic process |
|  | 8 | **Glyma.11G004200** | Glyma.20G143400 | na | na | na |
|  | 9 | Glyma.11G062600 |  | na | na | na |
|  | 10 | **Glyma.01G135200** |  | na | na | na |
|  | 11 | Glyma.16G103900 |  | cellular metabolic process;steroid biosynthetic process | lignan biosynthetic process | na |
|  | 12 | **Glyma.18G220600** |  | cellular metabolic process;steroid biosynthetic process | brassinosteroid metabolic process; flavonoid biosynthetic process; regulation of brassinosteroid biosynthetic process | cellular metabolic process |
|  | 13 | **Glyma.13G285300** |  | na | na | na |
|  | 14 | **Glyma.02G307300** | **Glyma.14G005700** | na | na | flavonoid biosynthetic process |
|  | 15 | **Glyma.18G285800** |  | na | na | na |
|  | 16 | Glyma.04G230400 |  | na | na | na |
|  | 17 | Glyma.10G295200 |  | na | na | na |
|  | 18 | Glyma.19G121600 |  | na | na | na |
|  | 19 | Glyma.05G195000 | Glyma.14G152700 | na | na | na |
|  | 20 | Glyma.06G295400 | Glyma.13G298900 | na | na | na |

Supplementary Table 5. List of candidate genes for CMS biosynthesis.

| Gene name | log_2_FC | ^a^P | ^b^P | ^c^P | At homologs | Description |
| --- | --- | --- | --- | --- | --- | --- |
| Glyma.01G135200 | 5.9 |  |  | √ | AT4G31940.1 | cytochrome P450, family 82, subfamily C, polypeptide 4 |
| Glyma.02G005600 | 3.1 |  | √ |  | AT3G23250.1 | myb domain protein 15 |
| Glyma.02G134000 | 4.0 |  | √ |  | AT3G48700.1 | carboxyesterase 13 |
| Glyma.02G307300 | 5.6 |  | √ | √ | AT1G59960.1 | NAD(P)-linked oxidoreductase superfamily protein |
| Glyma.03G147700 | 6.3 |  |  | √ | AT5G42500.1 | Disease resistance-responsive (dirigent-like protein) family protein |
| Glyma.09G269500 | 5.2 |  | √ |  | AT2G45400.1 | NAD(P)-binding Rossmann-fold superfamily protein |
| Glyma.11G004200 | 3.2 | √ | √ | √ | AT1G47480.1 | alpha/beta-Hydrolases superfamily protein |
| Glyma.11G070500 | 3.9 |  | √ | √ | AT4G39230.1 | NmrA-like negative transcriptional regulator family protein |
| Glyma.13G284900 | 2.9 | √ |  |  | AT3G20660.1 | organic cation/carnitine transporter4 |
| Glyma.13G285300 | 5.7 |  | √ | √ | AT4G31940.1 | cytochrome P450, family 82, subfamily C, polypeptide 4 |
| Glyma.17G030400 | 6.7 |  | √ | √ | AT1G24020.1 | MLP-like protein 423 |
| Glyma.18G220600 | 3.9 | √ | √ | √ | AT2G45400.1 | NAD(P)-binding Rossmann-fold superfamily protein |
| Glyma.18G285800 | 4.1 |  | √ | √ | AT1G59960.1 | NAD(P)-linked oxidoreductase superfamily protein |
| Glyma.19G030500 | 6.6 |  | √ |  | AT5G39090.1 | HXXXD-type acyl-transferase family protein |

The candidate genes predicted by all three approaches are in red. All the DEGs are up-regulated in Daewonkong. The paralogs of the predicted genes are indicated by blue check mark.

^a^P: guide prediction using 41 genes in network

^b^P: guide prediction using 7 DEGs

^c^P: hub prediction using 7 DEGs


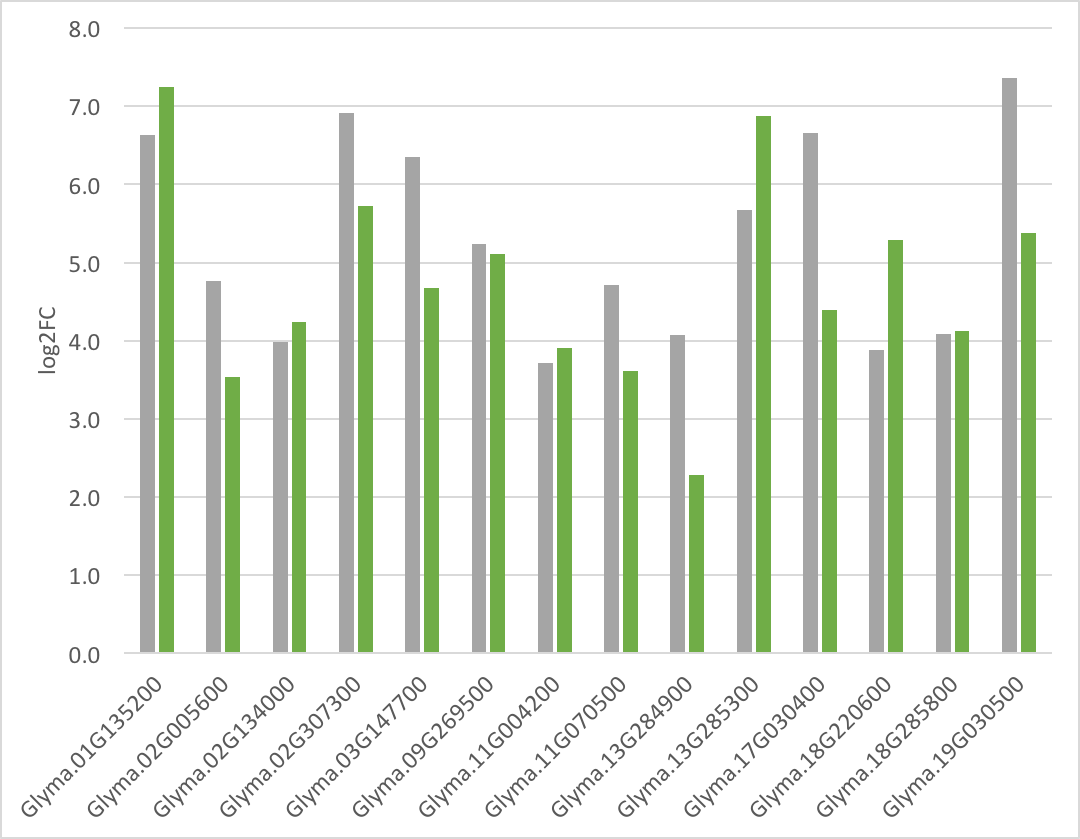


Supplementary Figure 5. Fold changes of expression levels in RNA-seq and qRT-PCR.

Fold changes of expression levels from RNA-seq and qRT-PCR are indicated by grey and green bars, respectively. All 14 genes are highly up-regulated in Daewonkong in both RNA-seq and qRT-PCR.
